# Thai Word Segmentation with a Brain-Inspired Sparse Distributed Representations Learning Memory

**DOI:** 10.1155/2023/8592214

**Published:** 2023-05-29

**Authors:** Thasayu Soisoonthorn, Herwig Unger, Maleerat Maliyaem

**Affiliations:** ^1^King Mongkut's University of Technology North Bangkok, Faculty of Information Technology and Digital Innovation, Bangkok, Thailand; ^2^University of Hagen, Chair of Communication Networks, Hagen, Germany

## Abstract

Word segmentation is necessary for many natural language processing, especially Thai language, that is, unsegmented words. However, wrong segmentation causes terrible performance in the final result. In this study, we propose two new brain-inspired methods based on Hawkins' approach to address Thai word segmentation. Sparse Distributed Representations (SDRs) are used to model the neocortex structure of the brain to store and transfer information. The first proposed method, THDICTSDR, improves the dictionary-based approach by utilizing SDRs to learn the surrounding context and combine with n-gram to select the correct word. The second method uses SDRs instead of a dictionary and is called THSDR. The evaluation uses the BEST2010 and LST20 standard datasets for segmentation words by comparing them with the longest matching, newmm, and Deepcut, which is state-of-the-art in the deep learning approach. The result shows that the first method provides the accuracy, and performances are significantly better than other dictionary bases. The first new method can achieve *F*1-Score at 95.60%, comparable to the state-of-the-art and Deepcut *F*1-Score at 96.34%. However, it provides a better performance *F*1-Score at 96.78% in learning all vocabularies. In addition, it can achieve 99.48% *F*1-Score beyond Deepcut 97.65% in case of all sentences being learnt. The second method has fault tolerance to noise and provides overall result over deep learning in all cases.

## 1. Introduction

Natural language processing (NLP) applications have grown exponentially, for example, sentiment analysis, information retrieval, text classification, machine translation, speech recognition, and question and answer. Some approaches request word level separately before processing in downstream tasks, for instance, using word embeddings for classification. Latin-based English language is easily tokenized into words by observing delimiter characters such as spaces, semicolons, commas, quotes, and periods. Unsegmented languages such as Thai, Chinese, Japanese, and Korean do not have explicit word boundaries to use delimiters to separate words. They require a specialized algorithm to find word boundaries before proceeding.

Thai word segmentation was developed firstly since 1981 and was divided into three types [1], namely, rule-based, dictionary-based, and learning-based techniques. Rule-based is created by hard coding. However, the language is complex and can only cover some existing rules or any unknown words that can introduce new rules. Dictionary-based uses a set of words from dictionaries by looking series of characters in the dictionary to find matches. The dictionary-based performance depends on the dictionary's size, the approach to handling unknown words, and the ambiguity that it founds multiple ways to segment a text. The easiest way to fix the ambiguity problem is by selecting the longest word. However, the performance is still low as the shorter word might be correct. Thus, another approach understands that the context of a word is mainly found in the learning-based technique.

The learning-based technique is learnt by marking word boundaries explicitly and using machine learning algorithms to build a model. The approaches include using the Hidden Markov Model (HMM) [2], Conditional Random Fields (CRF) [3], as well as Deep learning that is currently state of the art, such as Deepcut [4] and Attacut [5]. The advantage of the approach is that it has no requirement for dictionaries. The unknown word and ambiguity problem can be handled by using statistical characteristics. However, the drawback is that it requires a training data set and depends on the domain that is used to train, the size of training data, and labelling boundaries, which is a laborious task that takes time and effort.

This research proposes two new methods. The first method is based on the dictionary, and it can learn by using SDRs combination with n-gram, which is called THDICTSDR. The method adapts Hawkins's approach to Thai Word Segmentation problem by using Sparse Distributed Representations (SDRs) including the fact that it proposes a new encoder for Natural Language Processing (NLP) and combination with n-gram. The results show that neuroscience approach can also produce the accuracy performance comparable to the state of the art or deep learning approach. The second method, THSDR, uses SDRs instead of a dictionary to find words. This approach can improve fault tolerance to noise, which is particularly useful in applications where the data are not precleaned, such as Thai OCR.

It is important to note that this research is built upon a previous study [6] that introduced a new brain-inspired approach to address spelling check problems. This approach has been demonstrated to yield better results than deep learning methods, and it is also fault-tolerant to noise. Additionally, this approach is not limited to spelling check problems but can also be applied to Thai word segmentation in this research. Consequently, the research offers the following benefits:

1. There is no requirement to learn more from training data. The paper demonstrates that when the dataset includes more noise, the performance of deep learning models suffers. With deep learning models, the need to learn from a new error model requires retraining, which can be difficult to accomplish during working in an application. However, learning only from a dictionary or correct words is a simpler and more feasible alternative.
2. SDRs operate using bits, which makes it faster than numerical operations. For example, when finding similarities in a dictionary with 700,000 vocabularies, the processing time using numerical operations takes around 13 seconds. However, with SDR, the processing time is only 0.05 seconds.

In summary, this research has the following contributions:

1. Introducing a new combination method between dictionary-based and learning-based approaches that achieves accuracy performance comparable to state-of-the-art (Deepcut) and even outperforms it in some cases.
2. Proposing a new all-SDR approach that is capable of handling noise in situations where no training data exist for word boundaries. This approach is especially relevant for Thai OCR, which faces challenges associated with both spelling checks and word boundary identification simultaneously.
3. Demonstrating the advantages of the proposed method, including the ability to learn quickly by providing new vocabulary, which is not the case for the state-of-the-art that requires training data with explicit word boundaries for learning a new error model.

This paper is organized as follows: Section 2 presents related works, some of which are used to evaluate the new method.  Section 3 provides inspiration and background for this research.  Section 4 explains the concept of the new proposed approaches. Next, Section 5 demonstrates the evaluation method and results. Finally, in Section 6, a summary of this research is presented.

## 2. Related Works

The first rule-based [7] is created based on Thai grammar, and [8] improves the rules by using Thai spelling principles, and many rules are used in combination manners. The first dictionary-based [9] was proposed in 1986 using the dictionary combined with the longest matching technique by selecting the word with the longest length. If selecting cannot find the rest of the sentence, it will backtrack and find the next longest word. However, it will fail if the correct word is not the longest one or multiple unknown words are found. The maximal matching algorithm [10] was proposed to cope with the longest matching problem by finding all possible segmentation for a sentence and choosing one that contains the fewest words. Nevertheless, finding all possible words is a brute force method in a long sentence, and many candidates are generated. Therefore, the method does not guarantee the selection of the correct one and cannot determine the best one if candidates have the same number.

Another improvement of the dictionary-based is a trie structure that was proposed in 1991 [11]. Instead of keeping all words in a list, the method creates a structure like a tree to reduce the storage size and find words by moving in a tree node for each character. Thus, it can find the word via trie, which is faster than finding a word from a list. TLS-ART-MC [1] proposes a combination of Ranking Trie, Soundex and two-pass segmentation. Ranking trie is sorting the character node that depends on their frequency. A higher frequency node will be closer to the root node, and the node will be found first. Thus, it makes the trie structure smaller and performs faster. Soundex is used to cope with misspelling problems. The Soundex is designed for searching for an expected name with a different spelling. A word is converted to a code using rules; thus, the same code means the same word. Once the text passes segmentation from trie and Soundex, the segment words will be combined using rules based on Thai grammar.

Another approach is learning-based by using the statistical technique, a Viterbi-based, [2] to employ statistical information derived from grammatical tags. The idea is to find the path that gives the maximum probability. Moreover, [12, 13] propose using the word trigram with the part of speech. However, the method only captures its state and corresponding words, but the state might depend on something other than its following words or other adjacent words. In machine learning research, word segmentation is a classification problem that defines each character in the string as one of the binary classes, with the beginning of a word labelled “B” and an intraword character labelled “I.” These labelled characters and segmented words are trained with machine learning algorithms. Comparative research [14] is proposed to compare four learning-based algorithms that include Naïve Bayes (NB), Decision Tree, Support Vector Machine (SVM), and Conditional Random Field (CRF), the longest method and maximal matching. The result shows that the dictionary-based algorithms perform better than NB, Decision Tree, and SVM. However, the best result is shown by the CRF algorithm.

Instead of using segmentation words as a base, some research uses syllable or smaller group of characters that presents the performance better. In [15], the author uses two processes, namely, syllable segmentation and syllable merging. Firstly, the research defines about 200 syllable patterns and trigram statistics for syllable segmentation. Then, merging by finding possible sequence word segmentation from the dictionary and select the best word from the maximum collocation strength. In [3], the authors propose using minimum text units to extract the smallest units that constitute words and then using CRF to identify syllables and merge syllables by a set of rules. Another research proposes grouping Thai contiguous characters into inseparable units called Thai Character Clusters (TCCs) [16] that are defined by a set of rules. However, this research is provided for information retrieval to improve search accuracy.

The word segmentation state-of-the-art uses deep learning that can learn from data and error. A popular Thai word segmentation is Deepcut [4], which uses convolutional neural networks (CNNs) and shows that the accuracy from the experiment can achieve 96.57% and *F*1 at 96.34%. An improvement of Deepcut is Attacut [5] which uses syllable embedding as features together with character embedding. Even the accuracy and *F*1 of the research are not over Deepcut, but the processing time is at least 5.6*x* times faster than Deepcut. In [17], the authors provide word segmentation and POS tagging by jointing models of both tasks to improve overall accuracy. The word boundaries are produced first and then become an input for tagging. The input is character-level n-gram, and the next layer incorporates the n-gram features with their surrounding contexts by using bidirectional recurrent neural networks (RNNs). The accuracy results are above 90% on *F*1-Score.

## 3. Inspiration and Background

AI has been dramatically improved and developed today because of a machine learning approach called Deep Learning (DL), which produces impressive results by relying on backpropagation and mathematical optimization models. However, it is still far from the goal of creating AI at the level of human intelligence and unclear whether the current AI approaches can lead to this goal. Thus, instead of focusing on the current approaches, this paper aims to study the neuroscience approach and uses a solution called a brain-inspired method.

Although deep learning also replicates a function of neurons in the human brain, it has been steadily evolving since 1957. Its current behaviours are still not much different from how it started, which uses adjusting the weights between neurons as they learn. It is in contrast to the knowledge of neuroscience that has undergone further research and a vastly increased understanding of the neural system. This paper is not created from scratch but is based on Hawkins's approach or Hierarchical Temporal Memory (HTM) and adapting it to Thai word segmentation problem.

### 3.1. Hierarchical Temporal Memory (HTM)

HTM [18] is a theory that was initiated by Jeff Hawkins in the book “On Intelligence” [19] in 2004. It was built by reflecting the functioning of the neocortex from a neuroscience perspective. The HTM structure is similar to the neocortex as it is a uniform hierarchy and works in invariant representation characteristics. It can be separated into multiple layers, and each layer can break into a cortical column. A cortical column consists of multiple neural cells inside. Each sensory and neural cell connects by using synapses and dendrites. HTM can predict automatically by using Distal. Proximal is used to receive input signals and feedforward. It can learn by creating and strengthening connections with others if it is active together; that is called Hebbian Learning.

HTM provides a theoretical framework and basic mechanisms of how the neocortex works by inspiring and simplifying this research using SDRs, a basic form of each layer in the brain.

### 3.2. Sparse Distributed Representations (SDRs)

SDRs [20] are information storage and transfer information to feedforward and feedback in HTM. Information in an SDR contains “0” (Inactive) or “1” (Active) only. It is a large vector of bits with a small percentage active; this is how the brain works to reduce energy and inference with a small amount of activity. HTM uses Spatial Pooler for pattern recognition and Temporal Pooling to understand sequential learning. SDR is used as the structure memory of this research.

**Definition 1 .**

SDR is an n-dimensional vector of binary elements. SDR vector is as follows:

(1) $$\begin{array}{c} x=\left[b_{0},b_{1},\ldots ,b_{n-1}\right]\text{,} \end{array}$$

*w*_*x*_ is the number of elements in *x* that are active bits, “1.”

Overlapping is the number of bits that are 1 in the exact location, which is the determination of the similarity between two vectors. For example,

(2) $$\begin{array}{c} X=\left[1001000000000000000100000000000000001001\right]\text{,}\\ Y=\left[1000000000000001000000000000000000000001\right]\text{.} \end{array}$$

*X* and *Y* vectors have *n* = 40 and *w* = 5, overlap = 2 and sparsity is 12.5%, *s*=*w*/*n* (5/40).

Matching is the possibility of the number of unique SDRs as follows:

(3) $$\begin{array}{c} \left(\begin{array}{c} n\\ w \end{array}\right)=\frac{n!}{w!\left(n-w\right)!}\text{.} \end{array}$$

If *n* = 2048 and sparsity = 10% or *w* = 200, then the SDR space is 1.01 × 10^283^. This means the probability of two random vectors being identical is as follows:.

(4) $$\begin{array}{c} p\left(x=y\right)=\frac{1}{\left(\begin{array}{c} n\\ w \end{array}\right)}\text{.} \end{array}$$

Thus, with *n* = 2048 and *w* = 200, the probability of two identical random vectors is very close to zero.

#### 3.2.1. Union

A good characteristic of SDR is the union that can store multiple patterns in a single SDR using OR operation with vectors. Thus, it can reduce the size of storage kept in the brain. However, this approach can increase the probability of false positives. The probability of a false positive can be written as

(5) $$\begin{array}{c} p_{fp}={\left(1-{\left(1-\frac{w}{n}\right)}^{M}\right)}^{w}\text{.} \end{array}$$

For example, if *n* = 2048 and *w* = 200, storing *M* = 20 vectors, the chance of a false positive is 1 in 8.0 × 10^11^. However, increasing the number of union vector sets, *M*, the false positive can become saturated with “1” bits, and random vectors will mostly return a false positive match.

### 3.3. Encoder

HTM can handle any input by using the same algorithm because it uses an encoder to convert any signals into SDRs before sending them to HTM. Creating an encoder is no easy task that keeps important features passing to HTM. The encoder selection is important because it impacts the model's performance. This encoder process is the same as passing visual information from the retina to the neocortex.

Examples of encoding can be found in [21]. However, encoding in NLP is not mentioned but refers to cortical.io, which is a commercial api encoding to SDRs and only provides a concept in [22]. In [23], the authors use HTM for document categorization. It uses TF-IDF, finds Latent Semantic Indexing (LSI), and encodes numerical features into SDRs. In [24], the anomaly detection in system logs that uses GloVe word embeddings is described [25] and the numbers are encoded into SDRs.

As aforementioned, NLP in HTM commonly uses encoding numbers into SDRs. This paper proposes a new encoder for NLP that not only creates each character into a representation but also their connections are formed into representations.

## 4. Concept

### 4.1. Structure Memory

**Figure 1:**
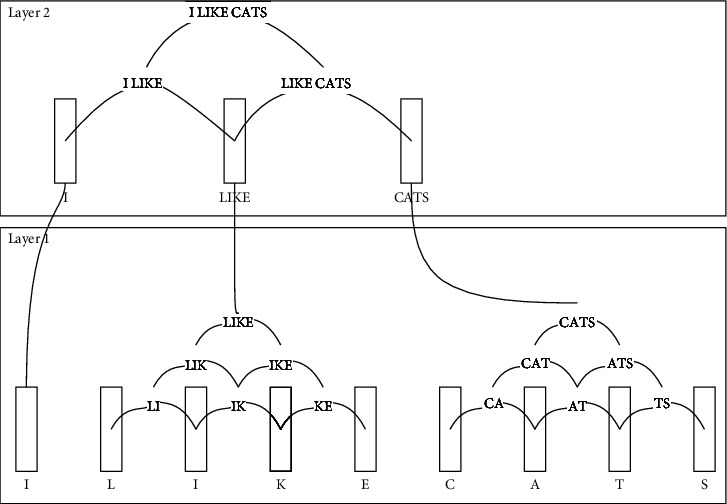
Forming connections for each representation and sequence pattern.

SDR is used to be the structure memory of this research. It can work in a hierarchy structure that one representation in a layer can connect to its layer and above or lower layer. For example, encoding a text is summarized in Figure 1; the first layer contains multiple cortical columns, and each column or representation is represented by an active “1” bit in SDR. A text can be encoded to any column depending on its encoding. This research proposes encoding by using a hash function that encodes not only each character to a representation but also a connection between representations is also a representation. The second layer is a representation of words and a sentence level where representations and connections work similarly to the first layer. This word and sentence information are kept in SDR, a large vector of bits with only a small number of actives based on the brain and inspired by Hawkins's approach.

One problem with word segmentation is that if multiple possible words are found, a basic approach is choosing the longest one. However, the longest word might not be correct; thus, one solution is understanding its surrounding context.

Training data, a word, and its surrounding context are encoded into an SDR using the concept in Figure 1. This means the algorithm learns its surrounding context and checks similarity values to determine which word can be segmented by selecting the highest similarity value. Estimating a similarity value can be found in the next section.

From Figure 1, “ILIKECATS” composes of “I,” “LIKE” and “CATS”; hence, word “I” contains its surrounding words, “LIKE” and “CATS.” Words “I,” “LIKE” and “CATS” are encoded into one SDR. For instance, the size of SDR is 10 bits. It composes of a zero vector [0 0 0 0 0 0 0 0 0 0]. The representation of “I” is hashing “I,” hash (“I”) %10 = 2, the output is [0 0 1 0 0 0 0 0 0 0]. In summary, the representation of “LIKE” can be hashed to “*L*,” “I,” “*K*,” “*E*,” “LI,” “IK,” “KE” and “LIK,” “IKE” and “LIKE”; the output is [1 0 1 0 0 1 0 0 1 0]. “CATS” works similarly to “LIKE.” Then each SDR will be unionized into one SDR representing “ILIKECATS” of the word “I.” Note that characters are not only converted into bits, but also their connections are formed using a hash function.

In this research, the number of active elements (*w*) and the number of bits or representations (*n*) are not specified. However, if its ratio is high, each SDR might not be separated from the others. Otherwise, the memory could have been used more efficiently. This evaluation is set *n* to 2048.

### 4.2. Similarity

Many machine-learning approaches use weights or floating numbers to predict their output. Instead, SDRs use bits or logical operations to process each representation. It can reduce complexity, including decreased processing time, as it is easy to manipulate because the CPU supports bit operations. Besides, the modern memory structure also supports keeping the information in bits; thus, it is easily adapted. The similarity estimation is calculated easily by finding overlapping bits, as shown in Section 3, using AND or XOR operations among SDRs. For example, “ILIKECATS” is similar to “ILIKEDOGS” as the number of overlapping is over “ILOVESONG.” The operation can perform very fast as it operates at a bit level.

## 5. Algorithm

### 5.1. Training

**Figure 2:**
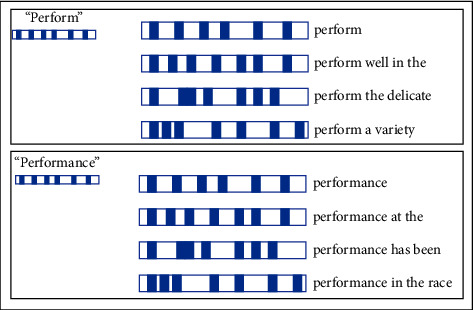
The structure of words and SDRs in THDICTSDR.

Each word in a dictionary is kept into a HashMap structure to link between a word and its SDR list. The word is encoded to be the first SDR for its list. Next, all words and its surrounding context are encoded into its SDR list. As Figure 2, the words “perform” and “performance” are kept into a map structure including SDRs of its word and surrounding contexts. The length of its surrounding context is set to a threshold (default = 16). The surrounding context can be suffix words or prefix words or both.

### 5.2. Matching

**Figure 3:**
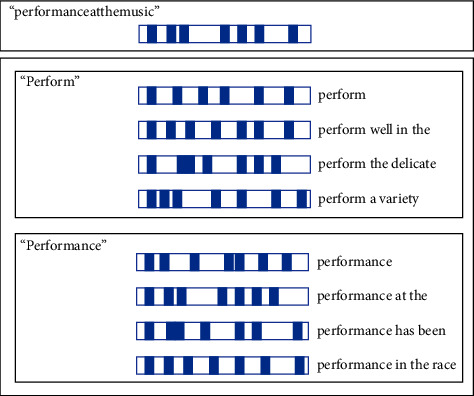
Example for surround text matching.

The THDICTSDR algorithm is based on a dictionary that searches for matches in a text. Firstly, it identifies a possible word list. If only one word is found, it selects that word. However, if multiple words are found, the algorithm chooses from their surrounding contexts, which are trained and stored in SDRs. For instance, as shown in Figure 3, we consider the text “performanceatthemusic.” The possible words could be “perform” and “performance.” Sample SDRs for the word “perform” in sentence forms include “perform well in” and “perform the delic,” while sample SDRs for the word include “performance” are “performance at *t*” and “performance has.” The similarity SDR value between “performanceatthemusic” and “performance at *t*” is the highest value, indicating that the word “performance” is the correct choice.

Similarly, in THSDR, the algorithm works like THDICTSDR, except instead of finding a possible word list; it looks for a match by comparing SDRs with words in the dictionary. The advantage of using SDRs is that they are fault-tolerant, meaning that even if some characters are missing or changed, the algorithm can still recognize the word. After identifying candidate words, the algorithm chooses from their surrounding contexts, which are trained and stored in SDRs, as in the previous example.

### 5.3. Handling Unknown Words

**Figure 4:**
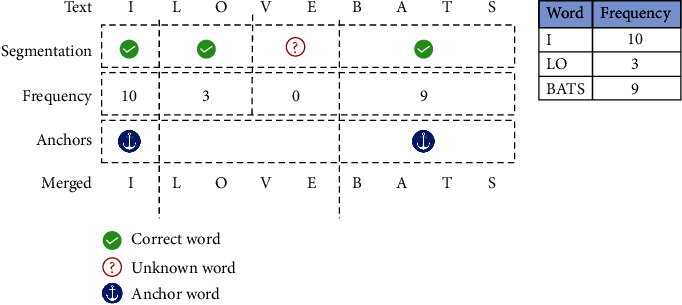
Handling unknown words by setting anchors and merging.

Handling unknown words, the author found that unknown words have a short length and low frequency. Another observation is that if an unknown word is found, there is a high possibility of segmenting words wrong previously; thus, it needs to backtrack. Hence, once it finds unknown words, it will set two anchor words between the unknown word by considering their length and frequency. For example, in Figure 4, known words and their frequency are [“I,” 10], [“LO,” 3], and [“BATS,” 9]. If a text is “ILOVEBATS,” the segmentation words are “I,” “LO,” “VE,” “BATS,” and “VE” is the unknown word. Nevertheless, we are sure that the words “*I*” and “CATS” are known words as they have a long length or high frequency. Thus, the algorithm set them as anchors. The unknown word “VE” searches neighbouring area and finds that “LO” has a low frequency; thus, “LO” and “VE” are merged into one word, “LOVE.”

#### 5.3.1. n-gram

We use n-gram to improve the accuracy performance; the author also found inconsistent segmentation words in training data. This problem is the same problem mentioned in [15] that suffers from a lack of clear definition, or even segmentation of the same person can be inconsistent. Thus, checking the co-occurrence value of the n-gram is performed. For example, for the two words “ice” and “cream,” if the frequency of “icecream” is more than the co-occurrence value of “ice cream,” then the two words “ice cream” will be merged into “icecream.” This research uses 2-gram to select the word.

## 6. Evaluation

This study evaluates the newly proposed method by comparing it with dictionary-based, longest matching, and newmm methods, which combine dictionary-based, maximum matching, and Thai character cluster [26]. The evaluation also compares the proposed method with Deepcut, which is the state-of-the-art approach. The evaluation is conducted using the Best2010 or Best and LST20 or LST Corpus datasets on an ASUS TUF A15 laptop with an AMD Ryzen 7 5800H, 8 CPU cores, 16 threads, 32 GB of RAM, and GPU RTX3060 6 GB. Best2010 [27] comprises 415 Thai documents, about 5.1 million words, and 104 k vocabulary, covering four domains, namely, articles, news, encyclopedias, and novels. The LST20 [28] Corpus, on the other hand, provides five layers of linguistic annotation, including word boundaries, POS tagging, named entities, clause boundaries, and sentence boundaries. It includes 3,164,002 words, 288,020 named entities, 248,181 clauses, and 74,180 sentences. Each dataset is used separately for training at 90% and testing at 10%.

Deepcut is trained from scratch by using this 90% training data set because the pretrain version of deep cut possibly included testing data. Likewise, THSDR bases on Lexitron Thai-Eng Dictionary and then learn from training data to create SDRs.

**Figure 5:**
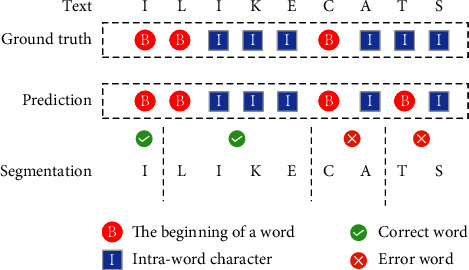
Word-level calculation for evaluation metrics.

Five metrics are used to evaluate the new model in word level, Precision, Recall, *F*1-Score, Intersection over Union (IoU), and processing time. Precision, Recall, and F1-Score can be calculated as an example in Figure 5 and equations 6–8.

Two parameters are evaluated; the first parameter was the length of the surrounding context, which was set to 8, 16, and 32. The second parameter was the size of the SDR, which was set to 1024, 2048, and 4096. During the experiment, a surrounding context length of 16 and an SDR size of 2048 were selected, as they resulted in the best performance.

(6) $$\begin{array}{c} Precision=\frac{the\;number\;of\;correct\;words}{the\;number\;of\;word\;predictions}\\ =\frac{TP}{TP+FP}\text{,} \end{array}$$

(7) $$\begin{array}{c} Recall=\frac{the\;number\;of\;correct\;words}{the\;number\;of\;words\;in\;the\;ground\;truth}\\ =\frac{TP}{TP+FN}\text{,}\\ IoU=\frac{TP}{TP+FP+FN}\text{.} \end{array}$$

(8) $$\begin{array}{c} F1-Score=\frac{2\times Recall\times Precision}{Precision+Recall}\text{,}\\ IoU=\frac{TP}{TP+FP+FN}\text{.} \end{array}$$

The equation above shows that TP (True Positive) represents the number of correctly identified word segments, while FP (False Positive) represents the number of misrecognized word segments. FN (False Negative) represents the number of unrecognized word segments.

In this evaluation, we tested the performance of the first method using a dictionary-based approach under three different scenarios. The first scenario involved the method only learning from the training data and a common dictionary. The second scenario assumed that the method could learn all the words in the sentences used for applications that provide interaction to users or that it had learned enough vocabulary to cover them. In the third scenario, the method had knowledge of all words and sentence connections. The second method was evaluated to demonstrate its fault tolerance to noise. We generated noise from the Best data set at four different levels: 1%, 3%, 5%, and 10% an measure the performances.

### 6.1. THDICTSDR Evaluation

The first evaluation, as shown in Table 1, indicates that the Precision, Recall, IoU, and *F*1-score on the best dataset for the longest matching and newmm methods are significantly lower than for Deepcut and THSDICTSDR. Although THSDICTSDR has higher recall performance than Deepcut at 96.89%, its precision, IoU, and *F*1-score have slightly lower performance. Additionally, the processing time of THSDICTSDR is higher than that of Deepcut due to its multiple rules to check. However, these results show that THSDICTSDR is comparable to state-of-the-art methods and offers a different approach. It should be noted that THSDICTSDR may have lower precision due to the unknown words problem, which even the proposed method cannot handle perfectly, resulting in some incorrect segmentation.

The second evaluation was conducted on another dataset, LST20, in Table 2. The results for THDICTSDR were similar to those in the first evaluation, with high recall but lower precision. While the performance of THDICTSDR was slightly lower than that of Deepcut, it still performed better than the longest and newmm methods.

Before correctly segmenting words, humans need to understand their vocabulary, and similarly, algorithms need to have a good grasp of the vocabulary for accurate segmentation. If any unknown vocabulary is encountered during segmentation, the software can notify the user and prompt for approval of a new word. This approach is different from the labelling of segmentation words used in deep learning for learning the error model. To validate if all the vocabulary was learned, the third evaluation in Table 3 was conducted. THDICTSDR outperformed Deepcut on recall, IoU, and *F*1-score, achieving 97.50%, 0.94, and 96.78%, respectively, on the best dataset, and 97.50% on Recall for the LST dataset. However, THDICTSDR still exhibits lower precision, IoU, and recall on the LST dataset due to its handling of unknown words.

In another case, the brain learnt correct words and word connections correctly and how the algorithm provides the performance results. In this case, the algorithm learns from training data and test on training data as assumption a human learnt all words and connections. As a result, in Table 4, THDICTSDR gives considerably *F*1-score at 99.48% on best and 99.37% on LST over Deepcut at 97.65%.

### 6.2. THSDR Evaluation

From the Table 5, THSDR provides high fault tolerance over Deepcut and deep learning approach in all cases.

### 6.3. Parameters

Table 6 evaluates SDR sizes of 1024, 2048, and 4096. The results indicate that there is not a significant difference in performance between the SDR sizes, including their processing time. Therefore, altering SDR sizes has minimal impact on overall performance. This suggests that increasing the SDR size to enhance the capacity for union operation in merging may be a viable approach to improve performance without sacrificing processing time.  Table 7 displays the results obtained by setting the word length threshold to 64, 32, 16, and 8. The findings suggest that increasing the word length can adversely affect performance as too many words are encoded into the SDR, leading to multiple matches. Conversely, reducing the word length can also have a negative impact on performance as it results in low surrounding context words.

### 6.4. Complexity and Time

Complexity and time can be broken down into two parts, namely, training time and prediction time. Training data are encoded into SDRs and stored in memory. Therefore, the complexity of training time is O (nh), where *n* is the number of training data points and *h* is the number of hash functions used. The complexity of prediction time is O (ns), where *n* is the number of training data points and *s* is the size of the SDR. Processing time reduction can be achieved through the use of union operations and parallel processing, as demonstrated in [6].

## 7. Conclusion

This research presents two new methods that use SDRs to replicate learning from the brain. Both methods exhibit higher accuracy than other dictionary-based methods. The first method also yields comparable results to the state-of-the-art Deepcut, and in some cases, even better. However, its processing time is still higher than other methods, and future research will aim to improve this aspect. The second method demonstrates good fault tolerance to noise, making it suitable for applications such as Thai OCR. This research is open-source and available at https://github.com/thasayus/thaiword-thsdr.

## 8. Future Work

There are many challenges and a lot of future work that can be done as follows.

1. Encoding SDRs for NLP currently relies on hashing between characters and their connections to reduce size and processing time. To improve performance, it may be possible to use syllable patterns instead of characters.
2. The processing time of the proposed methods is still slower than the state-of-the-art. One solution could be to use parallel processing and union vectors to speed up the computations [6].
3. The second method, THSDR, not only performs word segmentation but is also capable of correcting words in a hybrid manner for both word segmentation and spelling check problems. This hybrid approach has not been found in previous Thai language research. However, it falls outside the scope of this paper.
4. While the paper only employs SDRs from HTM, it would be valuable to explore the potential of other HTM techniques, such as the spatial pooler and temporal memory, for learning and predicting segmentation.

## Figures and Tables

**Table 1:** Word-level performance comparison on Best2010.

Method	Train data	Test data	Precision (%)	Recall (%)	*F*1-score (%)	IoU	Time (s/word)
Longest	Train	Test	77.01	67.05	71.68	0.56	3.95*E* − 05
Newmm	Train	Test	80.30	69.96	74.77	0.60	7.34*E* − 06
Deepcut	Train	Test	96.11	96.57	96.34	0.93	1.50*E* − 03
THDICTSDR	Train	Test	94.32	96.89	95.60	0.92	0.03

**Table 2:** Word-level performance comparison on LST20.

Method	Train data	Test data	Precision (%)	Recall (%)	*F*1-score (%)	IoU	Time (s/word)
Longest	Train	Test	80.60	72.06	76.09	0.61	4.20*E* − 05
Newmm	Train	Test	83.28	73.76	74.77	0.64	7.45*E* − 06
Deepcut	Train	Test	96.67	97.13	96.90	0.94	1.50*E* − 03
THDICTSDR	Train	Test	94.42	96.85	95.62	0.92	0.028

**Table 3:** Word segmentation performance on all learned vocabularies.

Method	Train data	Test data	Precision (%)	Recall (%)	*F*1-score (%)	IoU	Time (s/word)
THDICTSDR	Best train	Best test	96.08	97.50	96.78	0.94	0.03
THDICTSDR	LST train	LST test	95.48	97.50	96.40	0.93	0.03

**Table 4:** Word segmentation performance on all learned words and sentences.

Method	Train data	Test data	Precision (%)	Recall (%)	*F*1-score (%)	IoU	Time (s/word)
Deepcut	Best train	Best train	97.42	97.89	97.65	0.95	1.50*E* − 03
THDICTSDR	Best train	Best train	99.20	99.55	99.48	0.99	0.03
THDICTSDR	LST train	LST train	98.36	99.69	99.37	0.98	0.03

**Table 5:** Word segmentation performance with noise.

Method	Train data	Test data	Precision (%)	Recall (%)	*F*1-score (%)	IoU	Time (s/word)
Deepcut	Best	Noise 1%	92.99	93.50	93.25	0.87	0.002349547
THSDR	Best	Noise 1%	97.89	98.00	97.95	0.96	0.407567715
Deepcut	Best	Noise 3%	88.77	89.22	88.99	0.80	0.002298372
THSDR	Best	Noise 3%	93.44	93.70	93.57	0.88	0.408186889
Deepcut	Best	Noise 5%	84.62	85.00	84.81	0.74	0.002310482
THSDR	Best	Noise 5%	90.50	91.02	90.76	0.83	0.408712781
Deepcut	Best	Noise 10%	74.96	75.03	74.99	0.60	0.002285629
THSDR	Best	Noise 10%	79.29	82.00	80.62	0.68	0.426231586

**Table 6:** Word segmentation performance for varying SDR sizes.

SDR size	Word length	Precision (%)	Recall (%)	*F*1-score (%)	IoU	Time (s/word)
1024	16	93.47	96.82	95.12	0.91	0.03
2048	16	94.32	96.91	95.60	0.92	0.03
4096	16	93.64	96.82	95.20	0.91	0.03

**Table 7:** Word segmentation performance for varying word length.

SDR size	Word length	Precision (%)	Recall (%)	*F*1-score (%)	IoU	Time (s/word)
2048	64	83.36	93.53	88.15	0.79	0.4
2048	32	93.05	96.54	94.76	0.90	0.03
2048	16	94.32	96.91	95.60	0.92	0.03
2048	8	81.31	75.39	78.24	0.64	0.4

## Data Availability

The data used to support the findings of this study are available at https://aiforthai.in.th/corpus.php.
